# Enhanced taxonomy annotation of antiviral activity data from ChEMBL

**DOI:** 10.1093/database/bay139

**Published:** 2019-02-08

**Authors:** Anastasia A Nikitina, Alexey A Orlov, Liubov I Kozlovskaya, Vladimir A Palyulin, Dmitry I Osolodkin

**Affiliations:** 1 FSBSI “Chumakov FSC R&D IBP RAS”, Moscow, Russia; 2 Department of Chemistry, Lomonosov Moscow State University, Moscow, Russia; 3 Institute of Translational Medicine and Biotechnology, Sechenov First Moscow State Medical University, Moscow, Russia

## Abstract

The discovery of antiviral drugs is a rapidly developing area of medicinal chemistry research. The emergence of resistant variants and outbreaks of poorly studied viral diseases make this area constantly developing. The amount of antiviral activity data available in ChEMBL consistently grows, but virus taxonomy annotation of these data is not sufficient for thorough studies of antiviral chemical space. We developed a procedure for semi-automatic extraction of antiviral activity data from ChEMBL and mapped them to the virus taxonomy developed by the International Committee for Taxonomy of Viruses (ICTV). The procedure is based on the lists of virus-related values of ChEMBL annotation fields and a dictionary of virus names and acronyms mapped to ICTV taxa. Application of this data extraction procedure allows retrieving from ChEMBL 1.6 times more assays linked to 2.5 times more compounds and data points than ChEMBL web interface allows. Mapping of these data to ICTV taxa allows analyzing all the compounds tested against each viral species. Activity values and structures of the compounds were standardized, and the antiviral activity profile was created for each standard structure. Data set compiled using this algorithm was called ViralChEMBL. As case studies, we compared descriptor and scaffold distributions for the full ChEMBL and its `viral’ and `non-viral’ subsets, identified the most studied compounds and created a self-organizing map for ViralChEMBL. Our approach to data annotation appeared to be a very efficient tool for the study of antiviral chemical space.

## Introduction

According to the 2016 release of viral taxonomy by International Committee for Taxonomy of Viruses (ICTV), there were more than 3700 different viral species (1), and at least 210 of them were known to cause human diseases (2, 3). Only 9 viral diseases caused by a dozen of viral species may be considered as treatable by drugs, and only 90 antiviral drugs based on around 70 different small molecule compounds were approved for treatment by 2016 (4). Therefore, a serious unmet clinical need for new antiviral drugs is clear. Given a significant amount of antiviral activity data in public databases (5), it is attractive to use data mining approaches based on chemical space analysis to study and predict the antiviral activity spectrum for small molecule compounds (6). Nevertheless, this task appeared to be not as straightforward as it would seem.

A previous attempt to mine the antiviral chemical space was made by Klimenko *et al.* (7), who constructed the antiviral subset of ChEMBL by selection of assays using the keyword search in the public web interface, obtaining a total of 24 633 compounds. The application of the Generative Topographic Mapping (GTM) machine learning approach to this subset allowed to successfully classify the antivirals according to target viruses and spectra of antiviral activity (7, 8). Seven major activity classes of antivirals, corresponding to certain genera, were considered in this study, thus allowing further detalization of the GTM antiviral chemical space sketch.

**Figure 1:**
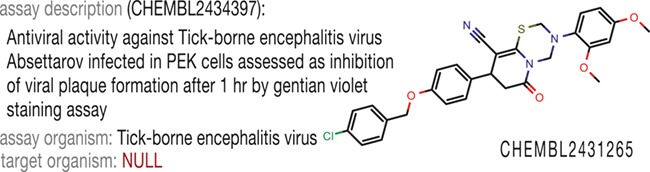
Example of incomplete data annotation in ChEMBL.

When we accessed ChEMBL (9) to find the information about antiviral activity against tick-borne encephalitis virus for compounds identified in our previous studies (10), we could not find these data through the biological taxonomy tree available in the web interface. Nevertheless, the structures themselves were present in the database, and the assay descriptions, as well as activity values, were correct, but the target organism field was empty (Figure 1). Thus, a deeper analysis of the database content was required to extract as many records relevant to antiviral activity as possible to build the antiviral chemical space.

The importance of the correct data annotation and standardization was highlighted in the field of quantitative structure-activity relationships (QSAR) and chemoinformatics model development and analysis (11, 12). In the framework of antiviral activity data analysis, two annotations are particularly important: target virus annotation and molecular target annotation. In the primary sources, such as experimental papers, representation of antiviral activity is greatly varied due to the variability of experimental methods, thus requiring an additional curation for some of ChEMBL data. The antiviral activity is usually assessed in limited throughput assays, e.g. plaque or cytopathic effect assays (13). A large amount of data was obtained using only these assays, and no further target mining was performed. These assay types are underrepresented in data ontologies; common viral reproduction inhibition assay formats fall into the unstructured branch `organism-based format' in BioAssay Ontology (14), used in ChEMBL, and specific branches for replicon-based assays are not developed at all.

The situation is additionally perplexed by the variability of mechanisms through which antiviral activity may be realized. These mechanisms may be divided into two large groups, utilizing host targets or viral targets, but molecular target information is usually not available for common antiviral assays with such endpoints as inhibition of viral reproduction or inhibition of viral replication. Thus, the molecular target annotation may be ignored on the first stage of antiviral data mining, and the correct annotation of assays to target virus species becomes the foremost task.

For sorting out the organisms, ChEMBL uses a simplified version of NCBI taxonomy (15, 16), which incorporates taxa from a wide range of sources, such as the published literature, web-based databases, data from sequence submitters, etc. (16). Being extended upon submission of new sequences in GenBank, this taxonomy contains separate entries for strains and isolates belonging to the same virus species, along with other name variants (16). A special disclaimer on NCBI taxonomy website states that `NCBI taxonomy database is not a phylogenetic or taxonomic authority and should not be cited as such’ (www.ncbi.nlm.nih.gov/Taxonomy/taxonomyhome.html/index.cgi?chapter=howcite). In contrast, ICTV taxonomy is developed by the expert community (17). This taxonomy is also constantly updated to reflect the scientific progress, and these updates are sometimes confusing and make the mapping of data from the scientific literature to different taxonomy releases complicated (18, 19). Nevertheless, for consistency of the studies, it is required to develop a scheme of mapping ChEMBL assays to ICTV taxonomy as the most comprehensive and expert-curated taxonomy. In 2008, a tool, named ORION-VIRCAT, was developed for mapping NCBI and ICTV taxonomies (20). This tool was based on a set of manually created annotation links for older taxonomy releases and thus cannot be directly applied to map current taxonomy releases.

In this manuscript, we present an algorithm for semi-automated extraction and curation of antiviral data from ChEMBL. Assay selection procedure employs the lists of relevant assay organism and target organism annotations and text mining of assay descriptions using dictionaries of virus-related terms. This advanced approach allowed us to extract 2.5 times more data points and compounds than ChEMBL taxonomy browser allowed (https://www.ebi.ac.uk/chembl/target/browser). Data points were annotated by virus taxa according to ICTV taxonomy; compound structures and activity values were standardized to obtain antiviral activity profile for each compound. This approach led to the most exhaustive and clearly annotated collection of publicly available antiviral activity data to date that we refer to as ViralChEMBL. Data visualization showed the features of this antiviral chemical space.

## Methodology

### Taxonomy representation

ICTV taxonomy is organized hierarchically with five possible taxonomic ranks (higher to lower): order, family, subfamily, genus and species (https://talk.ictvonline.org/taxonomy/w/ictv-taxonomy). The basic unit of the taxonomy is species, defined as `a monophyletic group of viruses whose properties can be distinguished from those of other species by multiple criteria’ (1, 21). Most species are grouped into genera, but higher ranks are undefined for many of them. Hereafter, only taxa names recognized by ICTV are italicized. Taxonomy structure was converted into a relational database (Supplementary data 1) organized as a tree with tables corresponding to the taxa (Table 1). On each level, one `unassigned’ entry was generated to deal with cases when exact ICTV species name could not be unambiguously assigned or for taxa not assigned to the higher levels by ICTV. A unique identifier was generated for each taxon (species_id, genus_id, subfamily_id, family_id and order_id) to be used for table connections.

**Table 1:** Taxonomy table statistics (placeholders included)

Table name	Taxonomy rank	Number of entries
ICTV.orders	Order	8
ICTV.families	Family	114
ICTV.subfamilies	Subfamily	237
ICTV.genera	Genus	770
ICTV.species	Species	3837

Additional field `pathogenicity flag’ (path_flag) was defined for each species. Integer non-zero value of this field is the reference number for the source of pathogenicity data (Supplementary data 2). These data were extracted from the biosafety documents (The Approved List of biological agents: http://www.hse.gov.uk/pubns/misc208.pdf) enhanced with other web resources (http://viralzone.expasy.org/678) and Google and Wikipedia searches and cross-checked with lists of pathogenic viruses compiled earlier by Woolhouse *et al.* (2, 3).

### Selection of assays

Data in ChEMBL are organized into a relational database, comprising separate interconnected tables. By proper selection of assays, compounds, for which biological activity was assessed in these assays, are extracted in a single step. Therefore, the main problem to obtain the antiviral activity data was to define the set of relevant assays properly.

Direct use of ChEMBL tax_id field values was not reasonable due to the inconsistency between NCBI and ICTV taxonomies. In general, tax_id values did not contain additional information compared to the data presented in the text annotation fields.

Four ChEMBL fields contain the information that can be used for text-based search of antiviral activity data: assays.assay_organism (organism used in an assay), assays.description (free text description of an assay), target_dictionary.organism (organism in which the intended molecular target of compounds tested in an assay resides) and target_dictionary.pref_name (name of the intended target protein). The analysis of plaintext values of assays.description field is the most complicated task. Taking into account that assay and target organism fields may contain non-virus-related values or placeholders even for the relevant entries (Table 2), we used assay description field as an additional data source to get as much relevant information as possible. Virus names may be also poorly standardized, e.g. in row 3 of Table 2, two different names for the same virus are used (its current species name is *Human alphaherpesvirus 1*).

**Table 2:** Examples of limited and contradicting information in ChEMBL fields: full information is available only in row 1, irrelevant values and placeholders are highlighted with bold typeface

assays.chembl_id	assays.assay_organism	assays.description	target_dictionary.organism	target_dictionary.pref_name	Reference
CHEMBL748873	Influenza A virus	In vitro inhibitory activity against influenza A neuraminidase using enzymatic assay	Influenza A virus [A/Puerto Rico/8/1934(H1N1)]	Neuraminidase	(22)
CHEMBL825021	**Homo sapiens**	Antiviral activity against envelope deficient HIV-1 in a single cycle replication assay (experiment 1)	**NULL**	**Unchecked**	(23)
CHEMBL662228	**NULL**	Inhibition of HSV-1 DNA polymerase in HSV-1 C42 plaque reduction assay	Herpes simplex virus (type 1 / strain 17)	Human herpesvirus 1 DNA polymerase	(24)
CHEMBL751813	Hepatitis C virus	Inhibitory activity against the hepatitis C virus NS3 protease was determined	**NULL**	**Unchecked**	(25)

**Table 3:** Virus-related entries in text annotation fields

Field	All values	Virus-related values	File
assays.assay_organism	3952	653	ao_list (Supplementary data 3)
target_dictionary.organism	2420	272	to_list (Supplementary data 4)

Since the data in different fields might contradict each other, all of them were analyzed separately in parallel. The lists of available values were extracted from the annotation fields assays.assay_organism and target_dictionary.organism. Then virus-related entries were manually chosen from them and directly mapped to ICTV taxa where possible (Table 3). The field target_dictionary.pref_name was not analyzed because it does not contain any new information about viral species. Data extraction from the table assays was performed via an SQL query using ao_list elements as the keys for assays.assay_organism. It gave the first set of relevant assays, which was added to ViralChEMBL.av_assays table*.* The second set of assays was extracted from assays table using to_list and target_dictionary table in a similar manner*.* Assays not present in ViralChEMBL.av_assays table were added to the table.

 The number of unique values in assays.description field was 965 591, not allowing manual analysis. Thus, automated procedures for data extraction were needed. A dictionary containing all taxa names from ICTV master species list (https://talk.ictvonline.org/files/master-species-lists/m/msl/5208) was compiled. To complement the dictionary with virus names present in ChEMBL, the records from ao_list and to_list were used. For viruses with available pathogenicity data (path_flag is not `NULL’), name variants (older names, synonyms and abbreviations) were added. The choice of mostly pathogenic viruses was dictated by ChEMBL data availability because antivirals were usually designed and tested only against pathogenic viruses, and for most plant viruses, phages, viroids etc., there were no antiviral data. However, ICTV taxa names and plant viruses' names present in ChEMBL fields allowed the extraction of some assays related to plant viruses (e.g. *Tobacco mosaic virus*). Full dictionary of virus names and name variants (older names, synonyms and abbreviations) contains 4814 entries and is available as Supplementary data 5.

**Figure 2:**
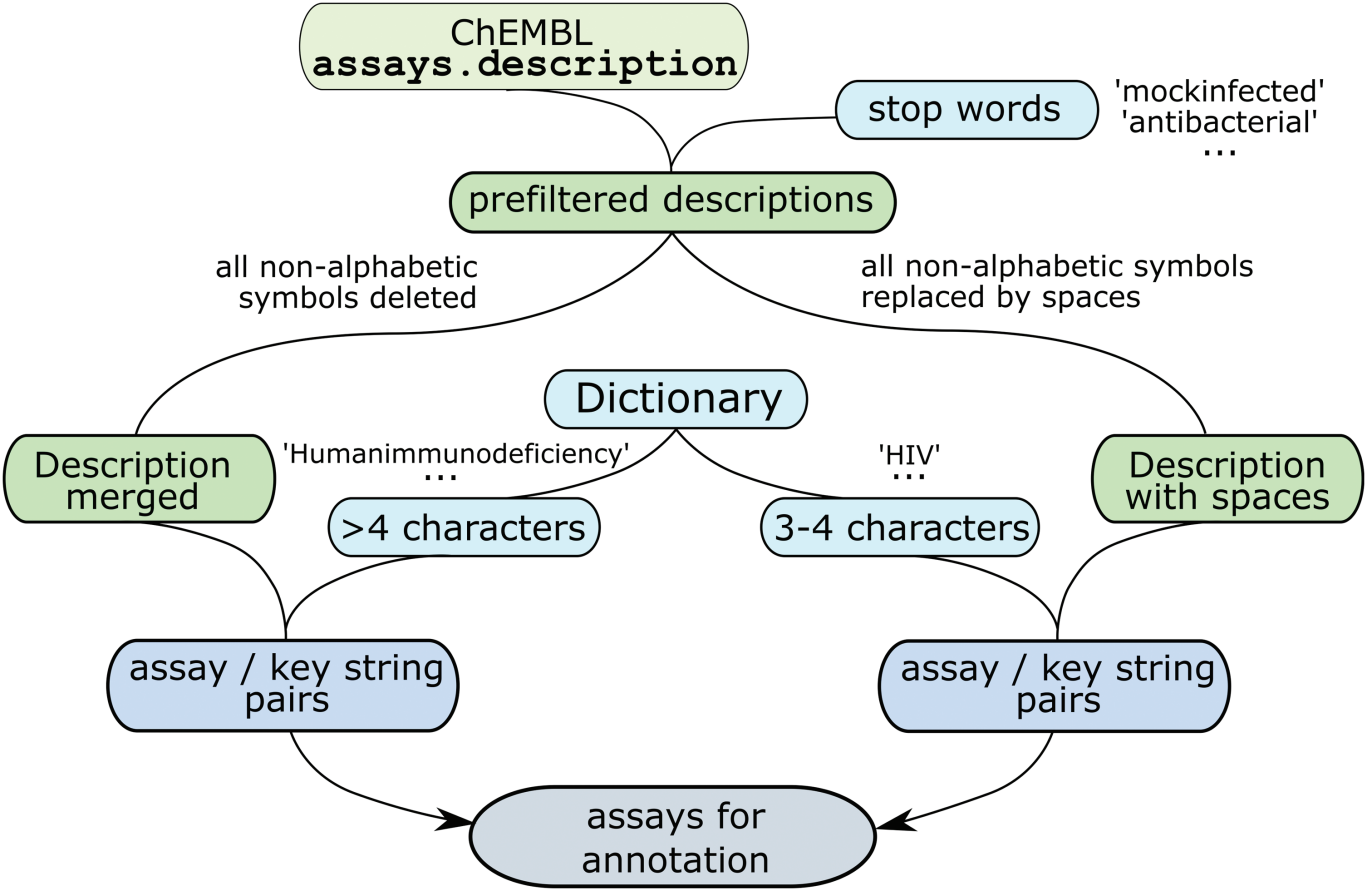
Procedure for filtering assays.description field values.

Dictionary of virus names and name variants was converted into a set of key strings to be searched in assay descriptions. Empirical rules, available as Supplementary data 6, were developed to maximize the number of extracted antiviral assays and to sort out irrelevant assays at the same time. All the assays were prefiltered using stop words. Then non-alphabetic characters, including spaces, were stripped from the key strings containing more than four alphabetic characters to obtain the minimal specific substrings. Non-alphabetic characters were stripped from assays.description field values as well and dictionary items of at least five characters in length were searched in these modified descriptions. Virus abbreviations of three to four characters flanked with spaces or line start/end symbols were searched in the modified assay descriptions, with all non-alphabetic characters changed to spaces (Figure 2). This search gave 101 174 pairs of assays and key substrings, with 559 virus-related substrings appearing at least once.

The substring dictionary was further extended with refined substrings to map the extracted assay descriptions to individual virus taxa. The shortest substrings were manually supplemented with alphanumeric symbols required to disambiguate viral species, then all items of this extended dictionary were mapped to the species identifiers from ICTV.species table. For example, substrings `hiv1’ and `hiv2’ were added to the extended dictionary with the aim to map data extracted using the substring `hiv’, where possible. If a substring could not be mapped to a species, `unassigned’ value of the corresponding taxonomy branch was used as a placeholder. The substring dictionary was supplemented with ao_list and to_list elements with assigned taxonomy identifiers. Substrings leading to extraction of large amounts of irrelevant descriptions [e.g. substring `icv’ corresponding to *Influenza C virus* is present in description `Compound was tested for blockade of locomotor activity in guinea pig, elicited by **icv** administered Sar9Met(O2)-SP’ (assays.chembl_id CHEMBL687446)] were dropped from the extended dictionary. This procedure led to 1192 pairs of key substrings and species identifiers.

### Annotation of assays

**Figure 3:**
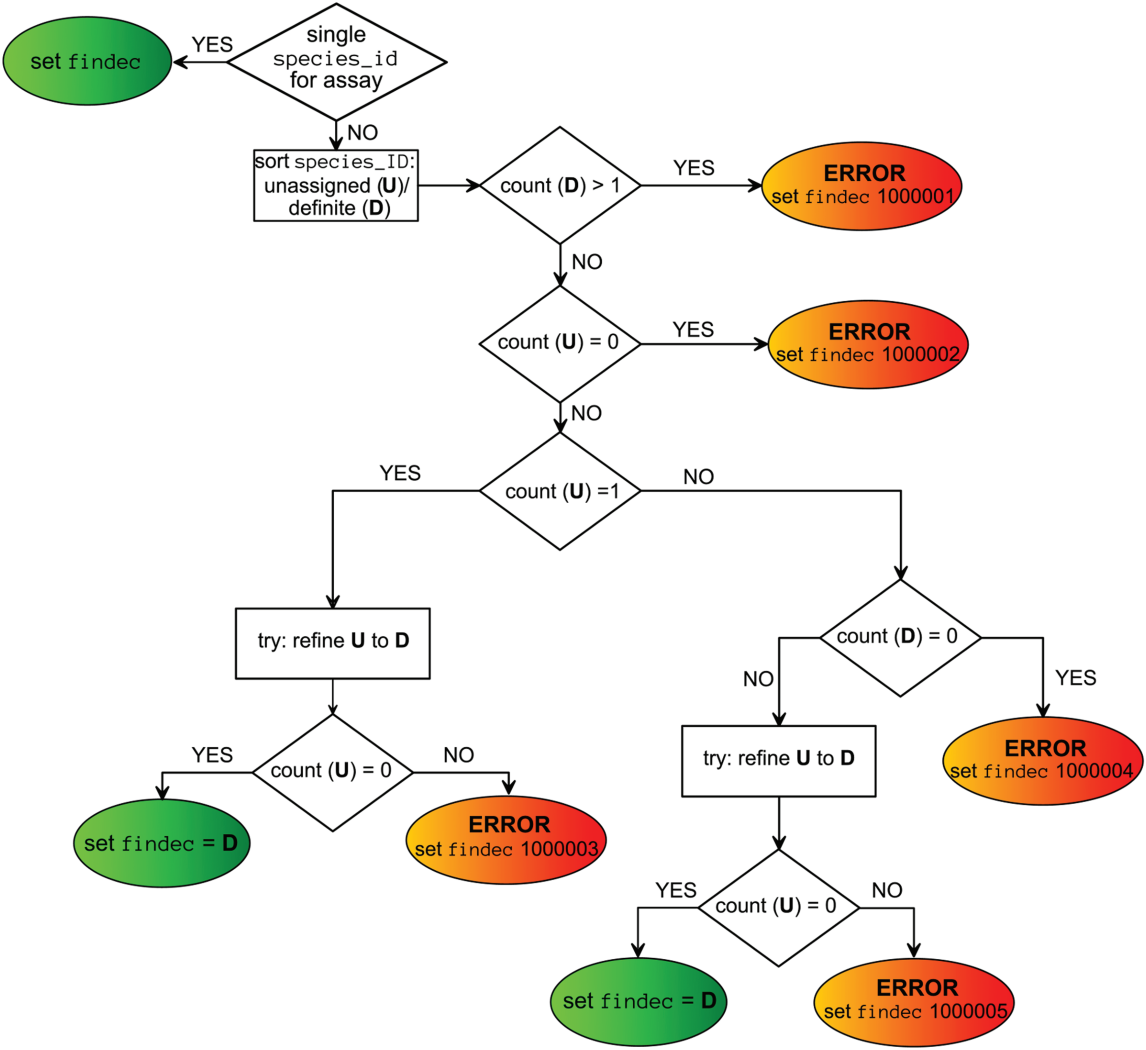
Decision-making scheme for the assignment of a viral taxon.

Viral taxon annotations were generated for ChEMBL assays extracted by values of assays.assay_organism, target_dictionary.organism and assays.description fields, independently. These annotations were put into the fields ao_tax, to_tax and dg_tax, respectively. Final taxon annotation (findec_a) was combined from these fields using a decision-making scheme (Figure 3) based on the choice of the taxon of the lowest level when taxa from different source fields did not contradict each other. If there was only one field containing viral taxon, this annotation was directly assigned as final. Mapping confidence score was assigned to each `findec_a’ based on rules defined in Table 4.

**Table 4:** Mapping confidence flag description

Mapping confidence (mapping_conf)	Fields with virus-related values			Number of assays
	assays.assay_organism	target_dictionary.organism	assays.description	
5	1	1	1	20 763
4	1	1	0	411
3^a^	1/0	0/1	1	5847
2^a^	1/0	0/1	0	377
1	0	0	1	9697
0	annotation inconsistencies (ERROR code in findec_a field)			283

^a^These mapping_conf values appear when only one of assay organism and target organism fields contains a virus-related value.

There were 316 assays with contradictory values of ao_tax, to_tax and dg_tax; these assays were put into a separate list for further analysis. The list was extended by assays extracted through text search using a set of strings, which could indicate non-antiviral assays [`antibacterial’, `antifungal’, `antitumor’, `vector’, `transformed’, `cell cycle arrest’, `apoptosis’, etc. (see Supplementary data 6 for the full list)], e.g. where virus was used for gene delivery. Obvious mistakes have been corrected during manual check-up of the list, and 283 complicated cases (1366 standard structures) will be solved through the literature sources; results will be reported elsewhere. Thus, most antiviral assays in ChEMBL were mapped to specific virus taxon records in ICTV.species table.

### Data standardization

Compounds tested in assays annotated as antiviral were extracted and standardized with ChemAxon Standardizer (JChem 14.11.3.0; ChemAxon, 2014, http://www.chemaxon.com) by stripping metal atoms, desolvation, removal of the smaller unbound fragment, aromatization, kekulization and tautomerization to the standard form (Standardizer XML file is available as Supplementary data 7). This sequence led to 260 866 unique standard structures. A unique identifier stdstr_id was assigned to each standard structure. ChEMBL IDs of initial compounds were retained and could be used for backwards compatibility and detalization of the data. The structure data were organized into the stdstr_mrgn table, which provided links between initial ChEMBL representations of compounds by molregno with stdstr_id, canonical SMILES representations std_smiles and molecular weight molw for standard structures. Entries with empty structures (851 unique molregnos) were excluded from this set.

Raw ChEMBL entries may contain numerous representations of activity inherited from original publications, and standardized activity values calculated during the database filling (pChEMBL) are not available for many of them. ViralChEMBL.av_activities table was created for viral-related assays based on the core structure of ChEMBL.activities table. Data from ChEMBL.activities were extracted for assays from ViralChEMBL.av_assays table by assay_id values and enhanced with fields molw, av_type, av_value and av_units, aimed to contain easily interpretable data from standard_type, standard_value and standard_units fields of ChEMBL.activities, respectively (Table 5). The list of all extracted field values is available as Supplementary data 8. Thus, ViralChEMBL.av_activities is connected to ViralChEMBL.av_assays through assay_id keys and to stdstr_mrgn through molregno keys. SQL script for activity data standardization is available as Supplementary data 9.

**Table 5:** ChEMBL activity data considered as interpretable

standard_type	standard_units	Number of entries
IC50	nM ug.ml-1^*^	67 208 4740
Potency	nM	335 316
Inhibition	nM %	177 14 848
Ki	nM	8587
EC50	nM ug.ml-1^*^	57 888 7496
MIC	nM	1843
ED50	nM um	2655 450

^*^Data with `ug.ml-1’ units were converted to nM.

Rules for binary classification of activity were developed for interpretable activity data. Activity flag av_act50 was assigned to each ViralChEMBL.av_activities entry using the rules provided in Table 6. For each stdstr_id–assay_id pair, a Boolean field was generated based on these rules to represent the activity of a compound in an assay. Activity data were summarized in sum_table, where each entry represents a stdstr_id–species_id pair. For each pair, p_50 parameter was calculated as *N*_active_/(*N*_active_ + *N*_inactive_), where *N*_active_ and *N*_inactive_ are numbers of entries for which a compound was classified as active or inactive, respectively. Antiviral activity profiles were constructed for each compound, represented by one-dimensional arrays of p_50 values, where array position corresponded to a single species.

**Table 6:** Activity data classification rules; av_act50 values

Active (1)	Inactive (0)
av_units = ‘nM’ AND av_value ≤50000 AND standard_relation = `=’ OR standard_relation = `<’	av_units = `nM’ AND av_value >50000 AND standard_relation = `=’ OR standard_relation = `>’
av_units = `%’ AND av_value ≥70 AND standard_relation = `=’ OR standard_relation = `>’	av_units = `%’ AND av_value <70 AND standard_relation = `=’ OR standard_relation = `<’
av_units IS NULL AND activity_comment LIKE `active’	av_units IS NULL AND activity_comment LIKE `inactive’

## Computational methods

### Databases

MySQL edition of ChEMBL v. 20 was used (9) (ftp://ftp.ebi.ac.uk/pub/databases/chembl/ChEMBLdb/releases/chembl_20/). Dump file was added to a local MySQL database. An internal structure of ViralChEMBL tables was based on the structure of the corresponding ChEMBL tables and complemented by taxonomy-related and -standardized values of the fields. The taxonomy database was created on the basis of the ICTV master species list (2014 v4 release) (https://talk.ictvonline.org/files/master-species-lists/m/msl/5208). Placeholder taxa `Unassigned’ were added to each level of taxonomy branches using SQL statement. DrugBank database (v. 5.0.7) was downloaded in structure-data file (SDF) format from web server (26) (https://www.drugbank.ca/).

### Data retrieval and annotation

Data retrieval was carried out using Python 2.7 in Spyder integrated development environment (https://www.spyder-ide.org/) and MySQL 5.7 Workbench interface (https://www.mysql.com/products/workbench/). On the first iteration, ao_list and to_list were used as keys for assay extraction. On the second iteration, assay description and assay_id primary keys were retrieved from ChEMBL via an SQL query. These descriptions were searched against virus name substring dictionary using a Python script (Supplementary data 6), and virus-related assays were extracted into a temporary file. All retrieved assays were mapped to ICTV taxonomy using the list of substring—species_id pairs (Supplementary data 10). For virus names containing another virus name as a substring (e.g. rhinovirus and *Inovirus*), a dictionary of corresponding substring pairs (Supplementary data 11) was used to avoid irrelevant mappings. Mapping was performed using a Python script available as Supplementary data 12. Final taxonomy identifier findec_a was chosen on the basis of ao_tax, to_tax and dg_tax identifiers using a script available as Supplementary data 13. If the identifiers represented different branches of ICTV taxonomy tree, the records were marked for the further manual check. Antiviral assay records from table ChEMBL.activities were extracted using an SQL query (primary key assay_id) and added to the table ViralChEMBL.av_activities; corresponding entries of ChEMBL.compound_structures were extracted via an SQL query (primary key molregno) and added to the table ViralChEMBL.compound_structures.

### Data standardization

ChEMBL compound structures were extracted as SMILES strings and saved in a comma separated values (CSV) file. Structures were standardized with ChemAxon Standardizer 14.11.3.0 (Supplementary data 7). Standard structure identifiers (stdstr_id) were assigned to initial structures. Molecular weight values for standardized structures were calculated in ChemAxon InstantJChem 17.1.30.0 (ChemAxon, 2017, http://www.chemaxon.com) and added to ViralChEMBL.av_activities table. Activity values were standardized to unified activity types and standard units (Table 5). DrugBank structures were standardized using the same procedure.

### Data analysis and visualization

DataWarrior v. 4.4.3 (27) was used for self-organizing map (SOM) creation. DataWarrior's fragment fingerprint FragFp was used for structure representation. Torus topology map of 50 × 50 neurons with `Gaussean’ neighborhood function was created using a fast routine for finding the best match.

Scaffold distributions were generated using DataWarrior `scaffold analysis’ function with Murcko scaffolds, defined as all plain ring systems of the molecule and their connections with each other. All substituents that do not contain ring fragments were deleted from the structure to obtain this kind of scaffold.

Molecular descriptors for histograms were calculated with ChemAxon JChem 16.8.29.0 (ChemAxon, 2017, http://www.chemaxon.com).

For the functional group analysis, the fully automated algorithm suggested in (31) was used. The algorithm is based on processing heteroatoms and their environment with the addition of some other functionalities. We used the implementation of this algorithm available in RDKit v. 2018.03.4 (http://www.rdkit.org).

## Results & Discussion

### Antiviral data

A compound may show antiviral activity mediated by viral or host targets. For purposes of antiviral data mining, an assay is considered to be relevant only if it assesses an organism-based or replicon-based antiviral activity or interaction with a viral protein. For numerous host proteins, participation in viral reproduction pathways is demonstrated, and, in certain cases, modulators of these proteins may show antiviral activity or even may be developed as antiviral drugs. Nevertheless, binding assays against these proteins cannot be considered for antiviral data mining if inhibition of viral reproduction by the compounds is not assessed in separate assays. On the contrary, despite binding or modulation of viral proteins *in vitro* do not necessarily lead to antiviral activity, viral proteins are often specific for viruses and do not have host analogs. In a classic target-based approach, these binding data form a core basis for further organism or replicon level antiviral activity studies and thus are relevant antiviral data.

**Figure 4:**
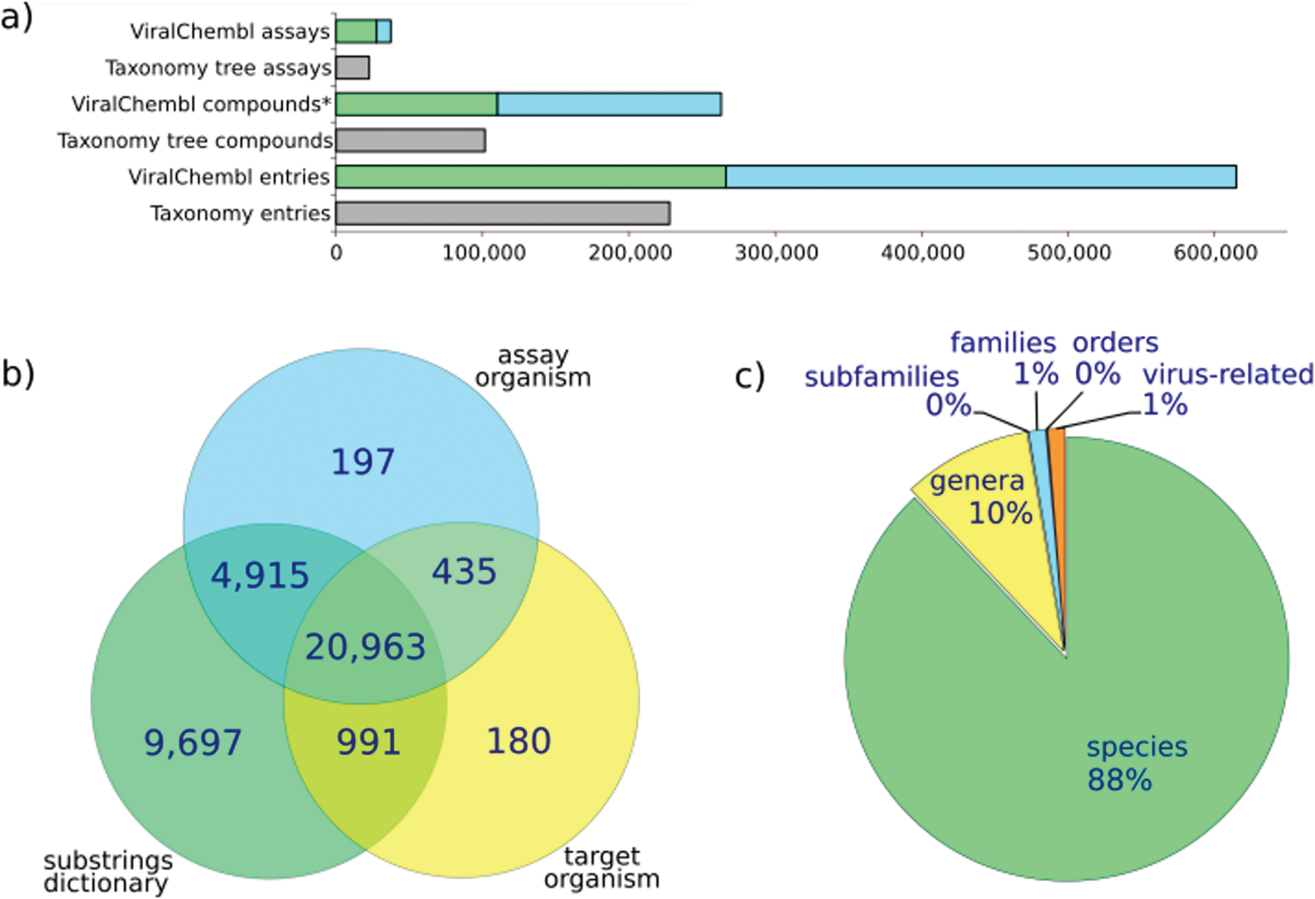
Assay statistics. (a) A number of assays, compounds (*, non-standardized) and entries extracted using ChEMBL web interface (taxonomy tree, accessed 12.04.16) or our procedure (ViralChEMBL); (b) number of assays extracted by each of used fields; (c) percent of assays mapped back to each taxonomy level.

The procedure of antiviral data extraction is designed with an aim to obtain as much data as possible and to annotate them on the fly, reducing the need for manual interventions. Statistics for antiviral assays extracted with our procedure is given in Figure 4. The core of the procedure is represented by an algorithm of mapping an assay to a viral taxon by the values of database fields. Extensive variation in virus name representation and not-always-obvious correspondence between common names and taxa of viruses make this mapping sometimes rather tricky. The situation when all relevant fields (av_assays.assay_organism, target_dictionary.organism and av_assays.description) contain values directly mappable to a certain species is the easiest, but a significant amount of assays presents the annotation conflicts or incomplete data. If an assay does not contain the virus-related terms in av_assays.assay_organism or target_dictionary.organism fields, it does not mean that this assay is not really virus-related. Text mining of assay descriptions allows enriching the collection with such assays.

Not every virus-related assay description may be mapped back to a single virus species. For example, strings `HIV’ or `human immunodeficiency virus’ cannot be mapped to species *Human immunodeficiency virus 1* (HIV-1) or *Human immunodeficiency virus 2* (HIV-2) without additional information. If the full assay description contains a substring allowing exact species mapping (e.g. `HIV-1’), this mapping is performed. However, if assay description mentions only `HIV’, placeholder species `Unassigned Lentivirus’ is to be used, placing an assay to the higher taxon branch (genus *Lentivirus* in this example). In more complicated cases a virus may be traced back only to the family level; this is typical for taxa with a rich history of changes and revisions, such as *Papillomaviridae*. In the case of annotation conflicts between different field values, the only reasonable decision was to raise an error. Several assays are definitely virus related, containing the `antiviral activity’ substring in the description, but no other clues are present to map them to any taxon, so they have all taxonomy levels set to ‘Unassigned’ (Table 7). There are 411 such assays that require further human insight and backward literature analysis. Nevertheless, their amount is negligible compared to the total number of annotated assays.

**Table 7:** Annotation procedure results

Assay ID	Description	Assay organism	Target organism	Viral annotations	Final decision
CHEMBL615372	Antiviral activity against 07/1 strain of VZV in HEL (human erythroleukemia) cells.	vericilla zoster virus^*^	Human herpesvirus 3	Assay organism, *Human herpesvirus 3*; target organism, *Human herpesvirus 3*; description, *Human herpesvirus 3*	*Human herpesvirus 3*
CHEMBL661557	In vitro activity against coxsackie B-4	Coxsackievirus	Coxsackievirus	Assay organism, unassigned *Enterovirus*; target organism, unassigned *Enterovirus*; description, *Enterovirus* B	*Enterovirus B*
CHEMBL695294	Inhibitory concentration against HCMV in plaque reduction assay	Human herpesvirus 1	Human herpesvirus 1	Assay organism, *Human herpesvirus* 1; target organism, *Human herpesvirus 1*; description, *Human herpesvirus 5*	ERROR CODE
CHEMBL698767	Inhibitory effect against influenza virus plaque formation at a concentration of 100 um	Unidentified influenza virus	Unidentified influenza virus	Assay organism, *Orthomyxoviridae*; target organism, *Orthomyxoviridae*; description, *Orthomyxoviridae*	*Orthomyxoviridae*

^*^As in ChEMBL. Correct name is Varicella zoster virus

There are six types of assays in ChEMBL 20: binding (B), functional (F), ADME (A), toxicity (T), physicochemical (P) and unknown (U) (28). This classification is based on the type of measured effect: for a binding assay, the measured value is related to the binding of a compound to a molecular target; for a functional assay, a particular biological effect caused by a compound (cell death, antiviral activity etc.) is measured; and ADME includes effects of compound metabolism, pharmacokinetics and pharmacodynamics. Functional assays comprise ~70% of all assays in ViralChEMBL and 40% in ChEMBL. We attempted to check the correctness of available assay attribution to B and F classes in the ViralChEMBL subset and found that this attribution is usually correct. Nevertheless, in this study, we made no distinction between binding and functional assays. A deeper analysis of assay types will be performed in future studies.

### Profiling of antiviral activity for ChEMBL compounds

**Figure 5:**
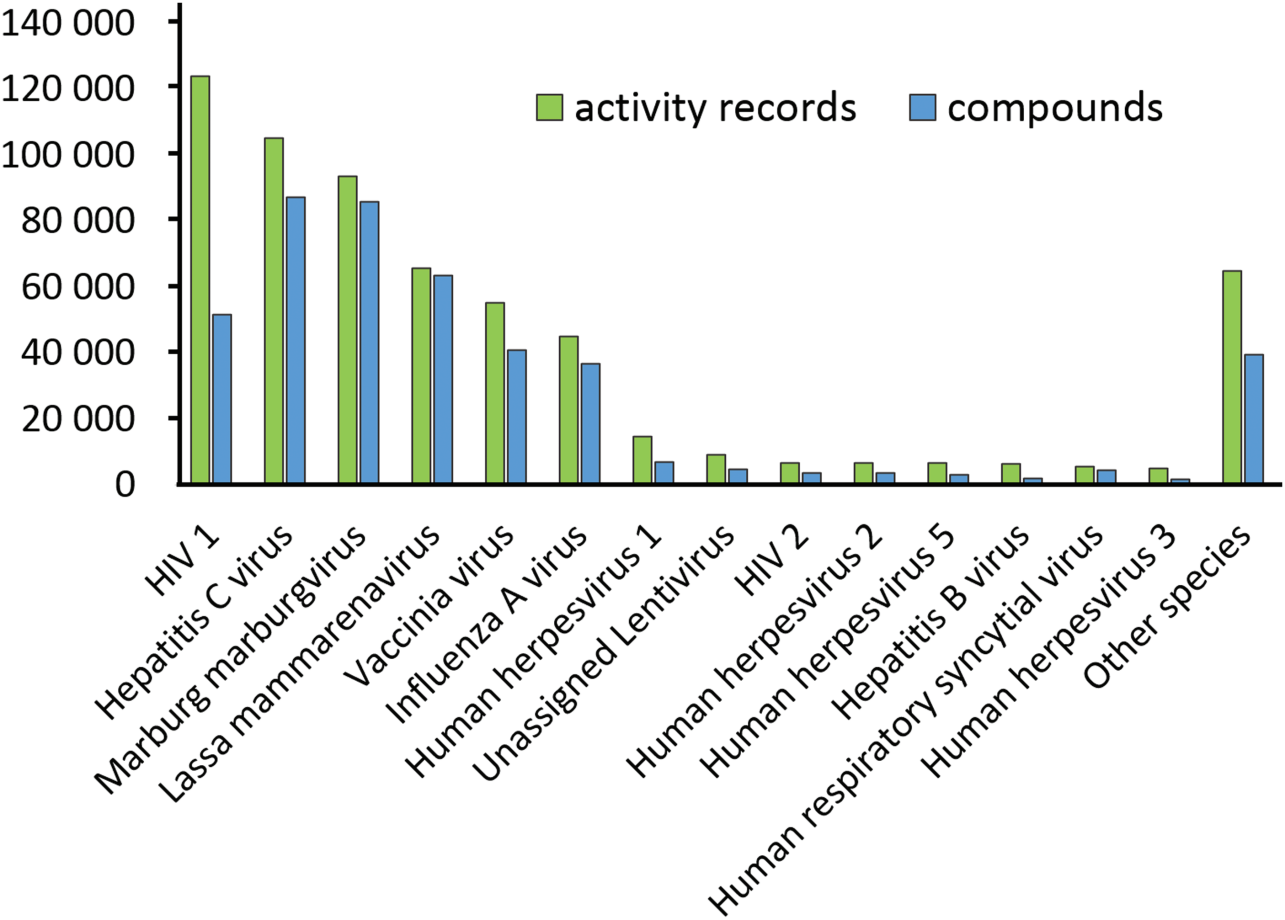
Statistics for individual activity entries (green) and standardized compounds (blue) mapped to virus species.

All compounds linked to assays marked as antiviral on the previous step are extracted and standardized. Standard structures form the centers of the nests comprising all their ancestors. Antiviral assays are mapped to these standard structures, and, for each standard structure, there is a list of assays where it has been tested (some examples are given in Table 8). Original identifiers (molregno) were preserved, and any additional information for these compounds may be easily extracted from ChEMBL. A list of tested compounds was created for each virus species (statistics are given in Figure 5 and Table 9). This system of interactions defines an enhanced subset of ChEMBL that we named ViralChEMBL.

**Table 8:** Compounds with the largest number of linked assays

Structure	Name	**Number of**		
		**species** ^*^	**genera** ^*^	**activity records**
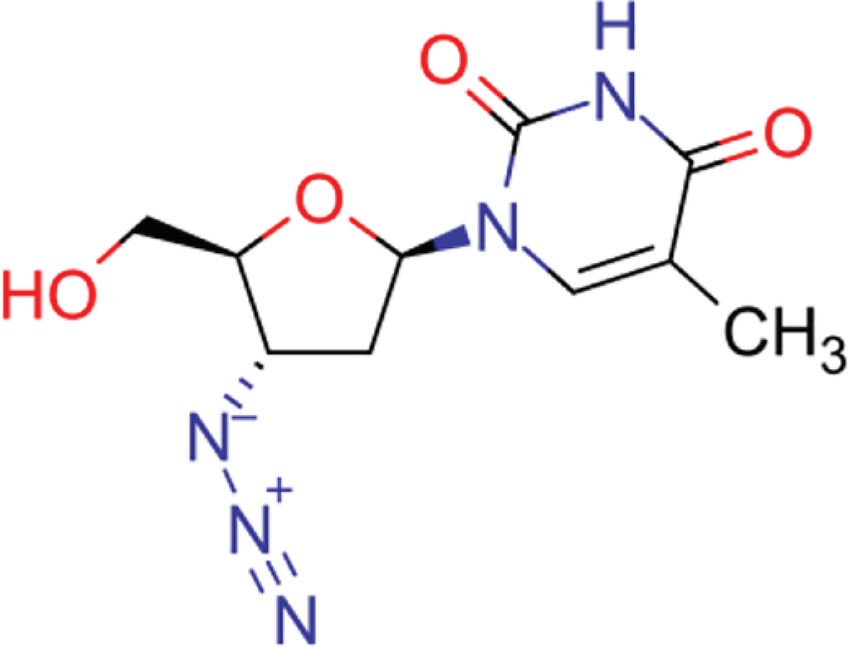	zidovudine	27	17	1739
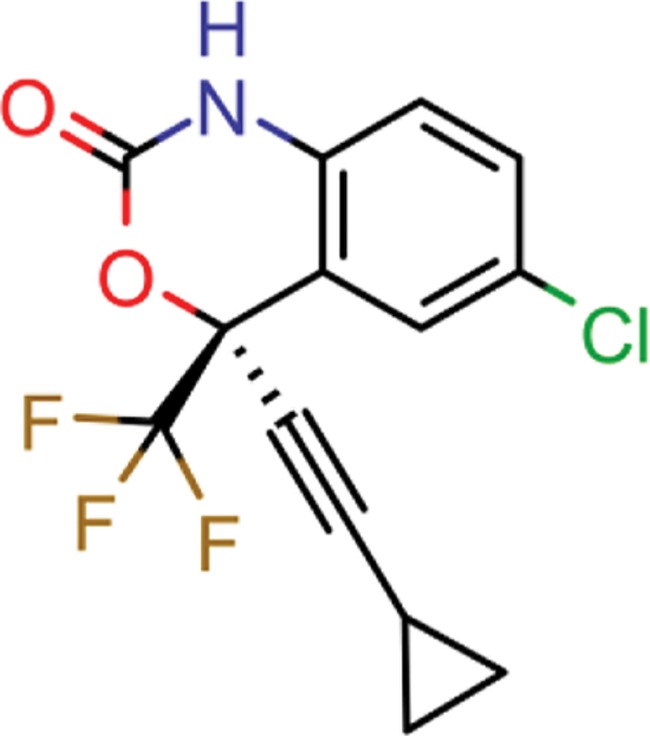	efavirenz	8	5	1612
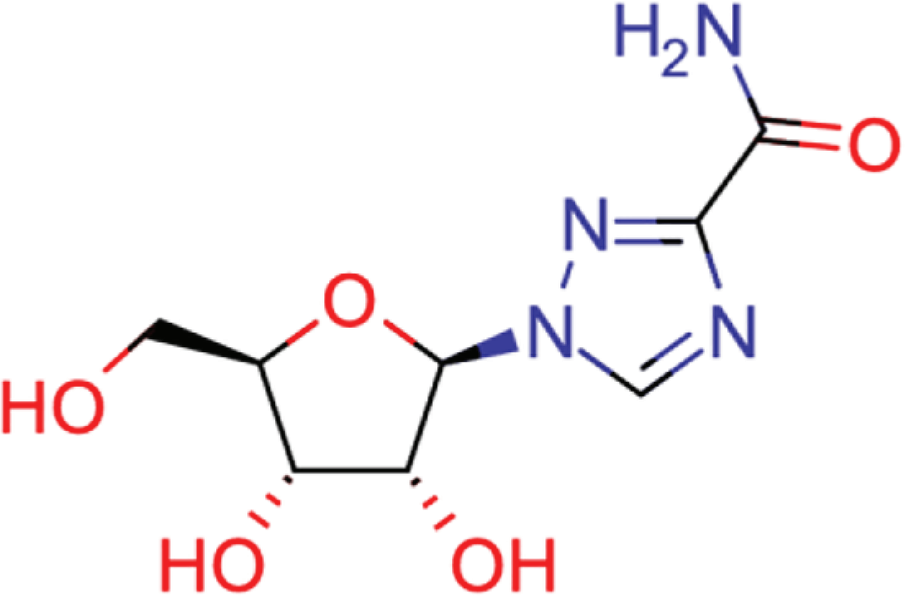	ribavirin	36	17	1517
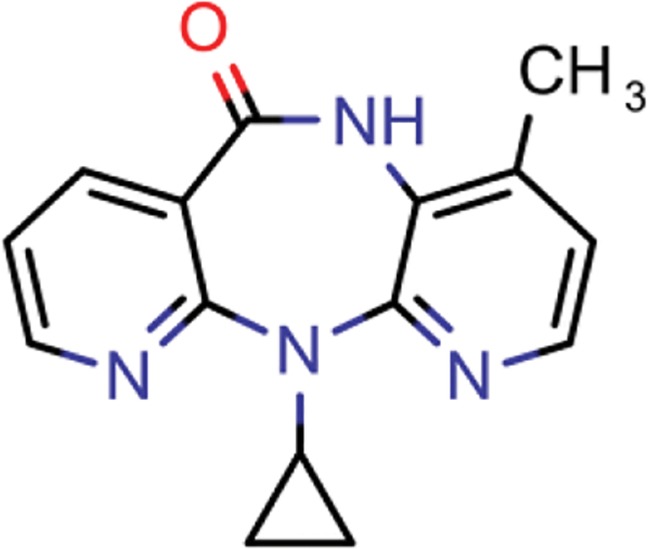	nevirapine	7	5	1514
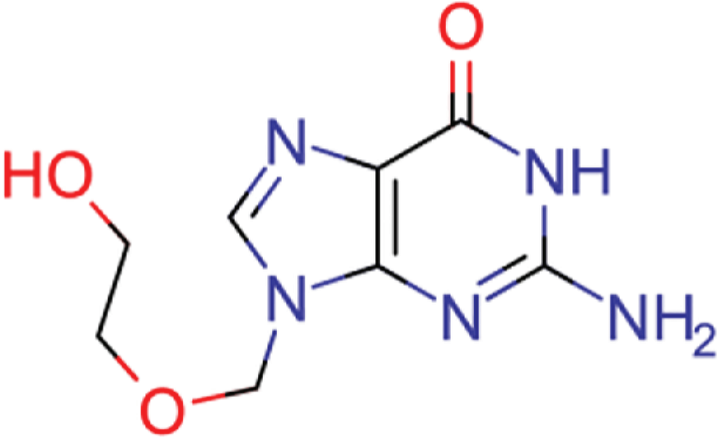	acyclovir	40	28	1459
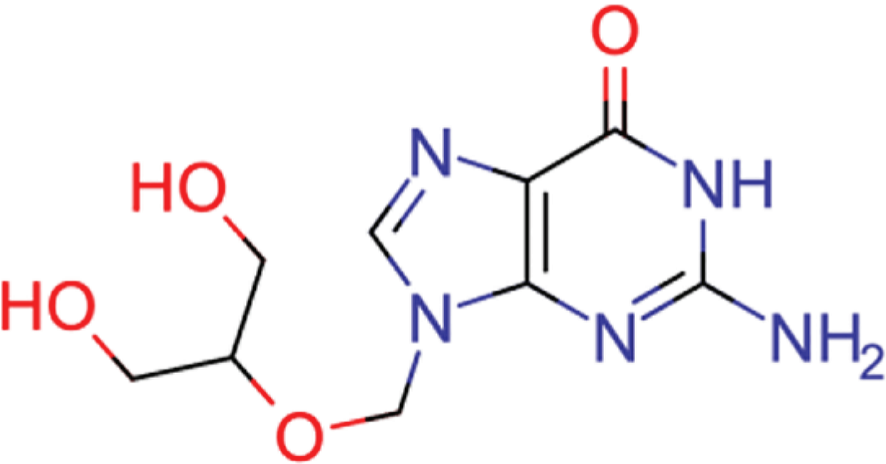	ganciclovir	35	27	1198
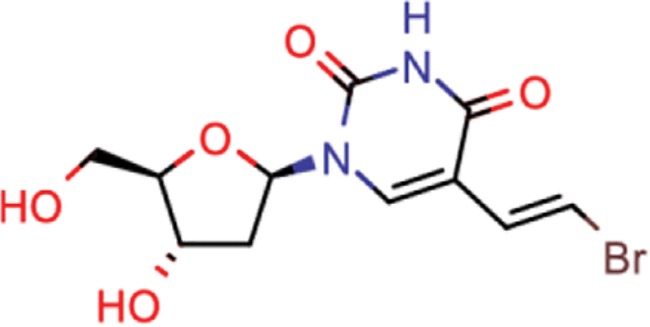	brivudine	33	23	985
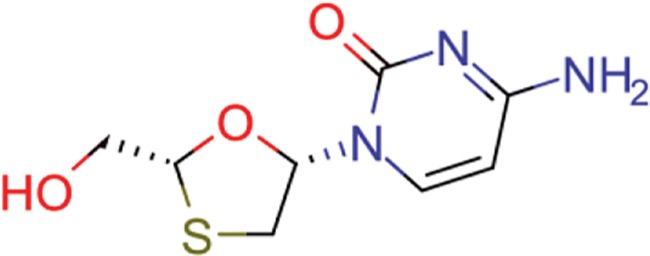	lamivudine	18	13	704
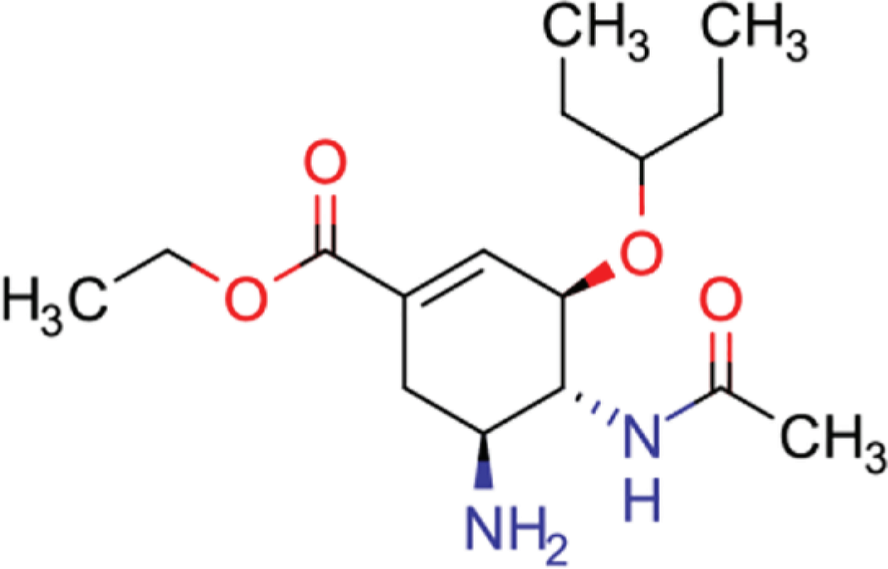	oseltamivir	22	19	646
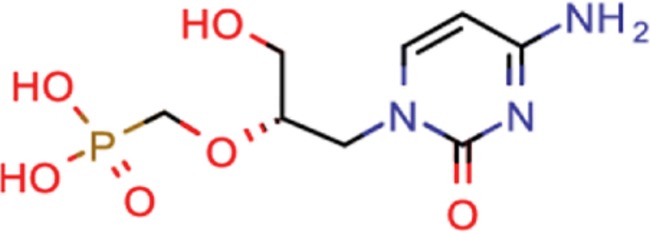	cidofovir	43	25	602

^*^Including ‘Unassigned’.

**Table 9:** Number of assays assigned to each virus species

#	Species	Assays
1	*HIV-1*	12 760
2	*Hepatitis C virus*	3274
3	*Human herpesvirus 1*	2417
4	*Influenza A virus*	2112
5	Unassigned *Lentivirus*	1789
6	*Human herpesvirus 2*	1215
7	*Hepatitis B virus*	1064
8	*Human herpesvirus 5*	1003
9	*Human herpesvirus 3*	727
10	*Vaccinia virus*	710
11	*HIV-2*	645
12	Unassigned *Enterovirus*	535
13	*Enterovirus B*	498
14	Unassigned *Vesiculovirus*	467
15	Other species	7818

**Figure 6:**
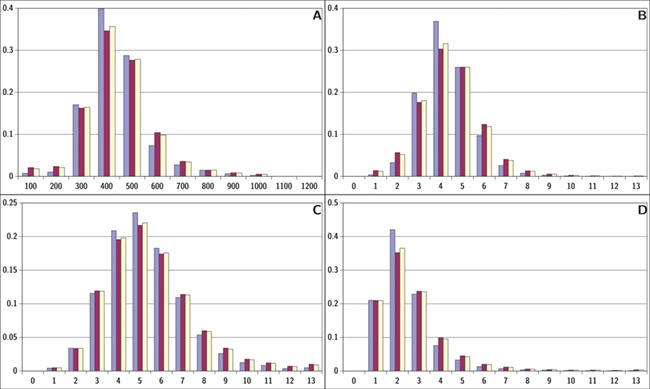
Descriptor distributions for ViralChEMBL (blue), non-ViralChEMBL (purple) and ChEMBL (yellow). (A) Molecular weight, (B) ring count, (C) hydrogen bond acceptors and (D) hydrogen bond donors.

A large amount of individual activity measurements for common broad-spectrum antivirals is quite expected, but even more data points are available for HIV-1 non-nucleoside reverse transcriptase inhibitors efavirenz and nevirapine. They were used in numerous HIV reverse transcriptase binding and functional anti-HIV assays as standards. Attempts to repurpose these molecules, if any had been performed, did not find their way to ChEMBL. On the contrary, typical broad-spectrum nucleoside analogs are often the first line of testing and treatment for emerging viruses and in drug discovery programs targeting specific viruses. Ribavirin is definitely a Jack of all trades and master of few in this field, given its low activity in most cases.

A `testing fingerprint’ for each ViralChEMBL compound is a 1D numeric array, elements of which represent the number of activity measurements against each virus species for the compound. Heatmap visualization of these testing fingerprints (Supplementary data 14) shows that the majority of the compounds were tested only against several most common viruses. This heatmap substantiates the need for an extension of efforts on testing new compounds against different viruses. For the majority of the compounds (255 883 of 260 520 compounds and 433 111 of 434 893 compound-virus pairs) there are no more than 10 data points in the database. For comparison, in a recent study of ChEMBL21, 4613 targets with at least 10 active compounds were identified (29).

Antiviral activity profiles were created using a more elaborate scheme, taking into account activity data type and the number of measurements. These profiles are bit strings, where the value of `1’ appears if the percent of interpretable activity measurements, where the compound was active against a certain virus species, is larger than 50% and `0’ otherwise. Reduction to interpretable activity values retains 85% of entries and is necessary to make the development of predictive models possible. The current implementation of ViralChEMBL does not take into account the mechanisms of action for the compounds, but preliminary classification models based on chemical space approaches may be useful for data analysis. Despite the mechanism of action classification exists in ChEMBL in the form of the confidence_score field, which ranges from 9 for direct assignment of a single protein target to 0 for unassigned targets, for more than 90% of ViralChEMBL assays, the single target is not assigned (confidence_score <8). Thus, for the moment, full-fledged annotation of the mechanism of action is not possible and further data curation is needed. On the other hand, a significant percent of non-interpretable data consists of assays with lower relevance to antiviral activity, e.g. ratios of activity and toxicity, which usually repeat data already available through activity and toxicity assays.

### ViralChEMBL versus ChEMBL

ViralChEMBL is a subset of ChEMBL containing presumably all compounds for which the antiviral activity or viral protein binding was measured at least once. Although these compounds may be classified as active/inactive against each virus species only separately, a global comparison of ViralChEMBL compounds with ChEMBL as a whole may be performed, as well as with the compounds never tested in virus-related assays.

**Table 10:** Top 10 most frequent scaffolds; the cells are coloured corresponding to the frequency of the scaffold in the ViralChEMBL dataset (from purple to red), gray background denotes scaffolds that are not present in the dataset; red frame highlights scaffolds specific to antiviral compounds

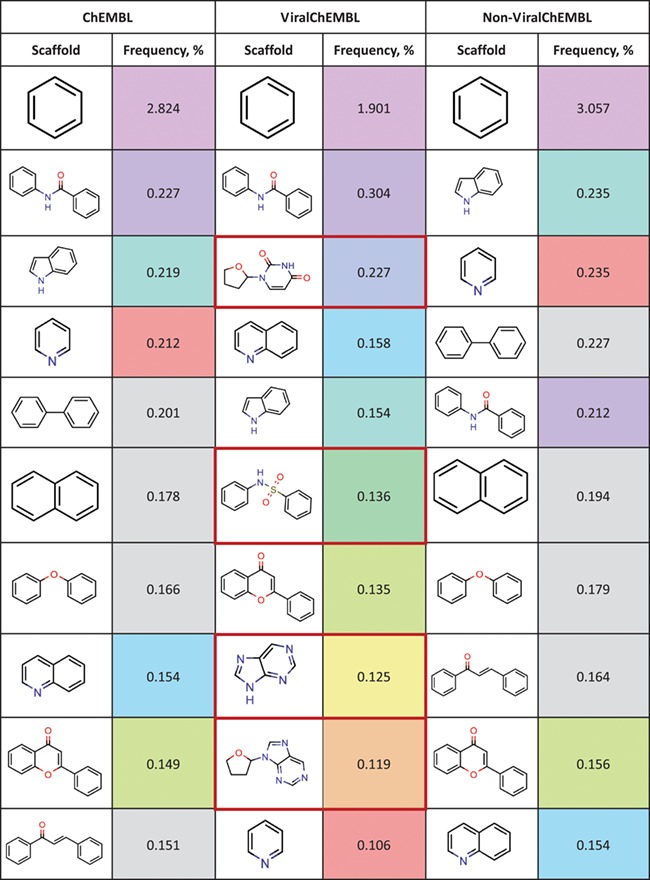

Distributions of simple descriptors for all databases did not show large deviations for any of them (Figure 6). It means that no specific selection rules are employed in the library design for antiviral HTS campaigns, and common Lipinski-compliant compounds are usually selected. On the contrary, scaffold distribution for databases is definitely different (Table 10). Whereas benzene ring is the most frequent scaffold in all sets, its frequency is different. ViralChEMBL is specifically enriched with nucleoside-like scaffolds because nucleosides are considered as privileged structures for antiviral drug discovery (30). Functional group counts in the sets also show some common and distinct patterns (Supplementary data 15). For example, aromatic carbon–nitrogen–carbon pattern is the most common for all the sets and shows almost the same frequency (present in 33.8% of ViralChEMBL compounds and 33.7% of non-ViralChEMBL compounds). On the other hand, aliphatic ether/alcohol pattern CO occurs in 14.7% of non-ViralChEMBL compounds (ranked third) but only in 10.9% of ViralChEMBL compounds (ranked fifth). More thorough analysis of structural features enriched in antiviral compounds based on these lists will be published elsewhere.

**Figure 7:**
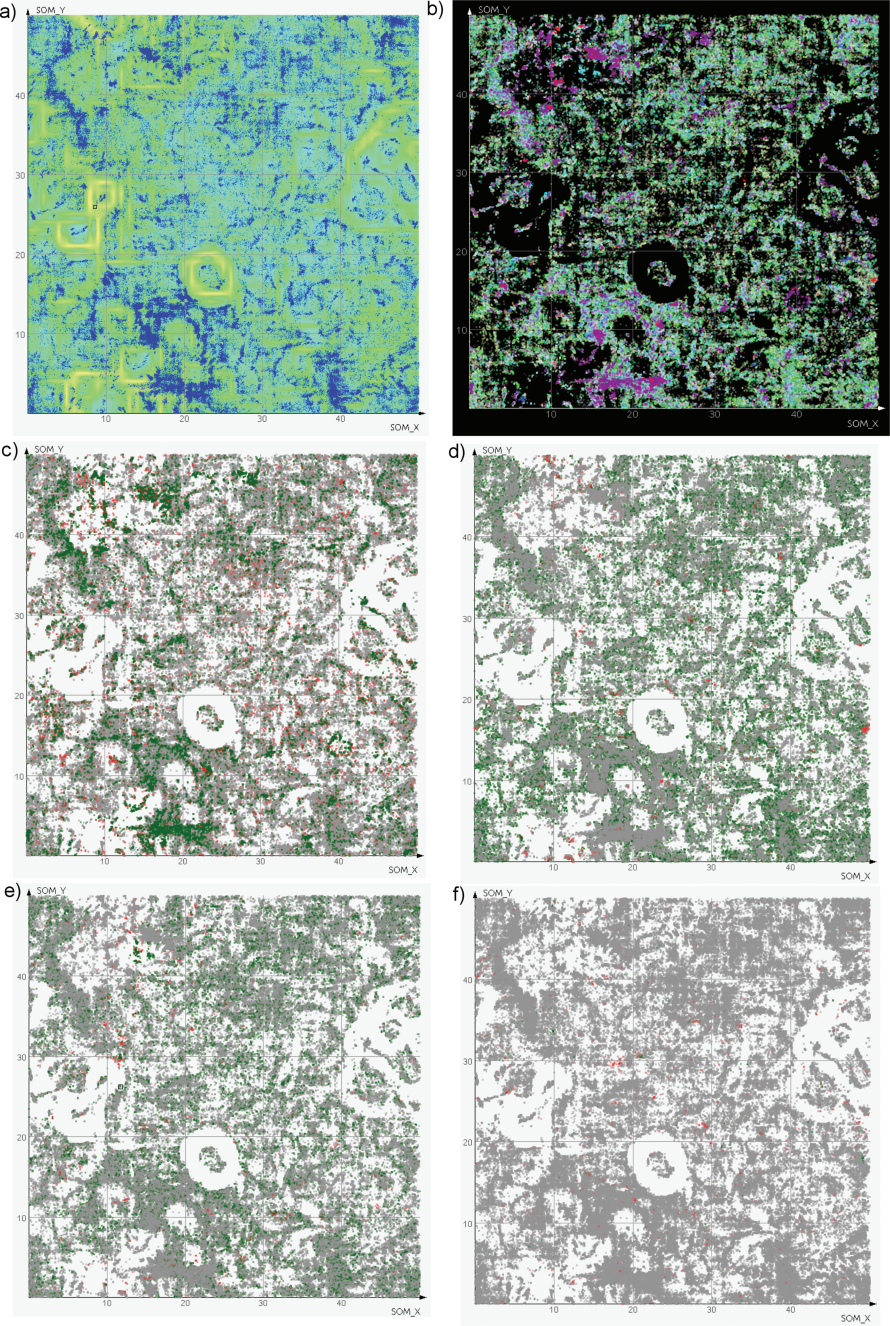
SOMs of antiviral chemical space. (a) All compounds (blue dots) with background colored according to neuron similarity. Greener areas correspond to high local similarity, yellow borders divide regions. (b) The same compound dots colored by virus family (color legend is available as Supplementary data 16). Note that compounds tested against more than one family (light yellow) are scarce. (c–e) The same dots colored by activity against a certain species: green, active; red, inactive; gray, not tested. (c) HIV-1; (d) *Hepatitis C virus*; (e) *Influenza A virus*; (f) *Dengue virus*.

**Figure 8:**
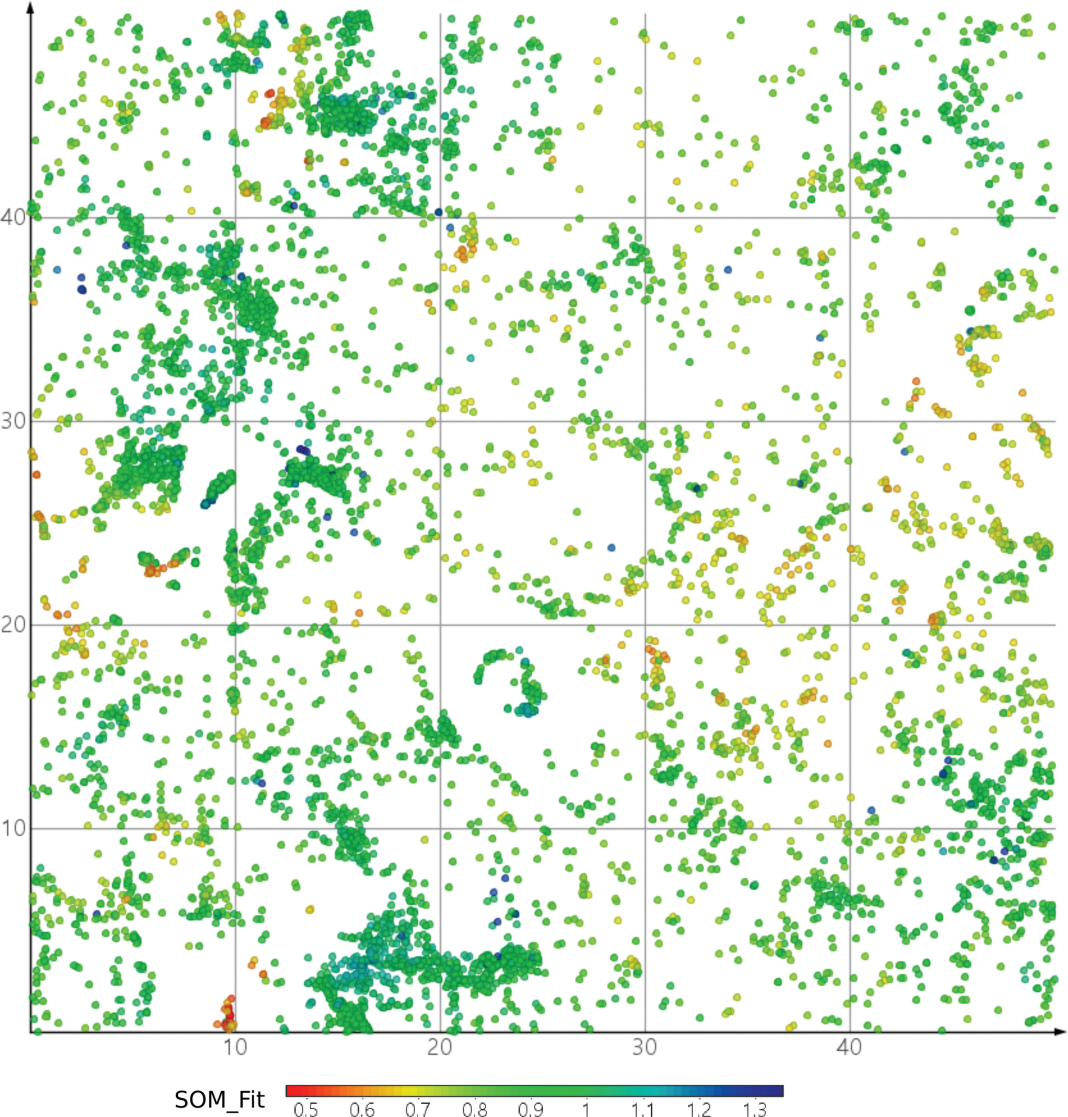
DrugBank mapped onto ViralChEMBL SOM. Dots are colored by SOM_Fit, higher values showing better fitness of projected compounds to their neurons.

**Table 11:** Examples of ViralChEMBL compounds close to DrugBank compounds

DrugBank compound	Name	Indications	ViralChEMBL compound	Activity	SOM distance	SOM_Fit	Tanimoto similarity
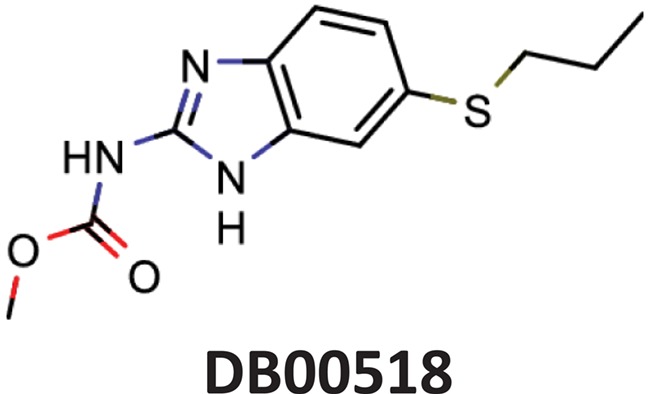	Albendazole	Parenchymal neurocysticercosis due to active lesions caused by larval forms of the pork tapeworm, *Taenia solium*; cystic hydatid disease of the liver, lung and peritoneum, caused by the larval form of the dog tapeworm, *Echinococcus granulosus*.	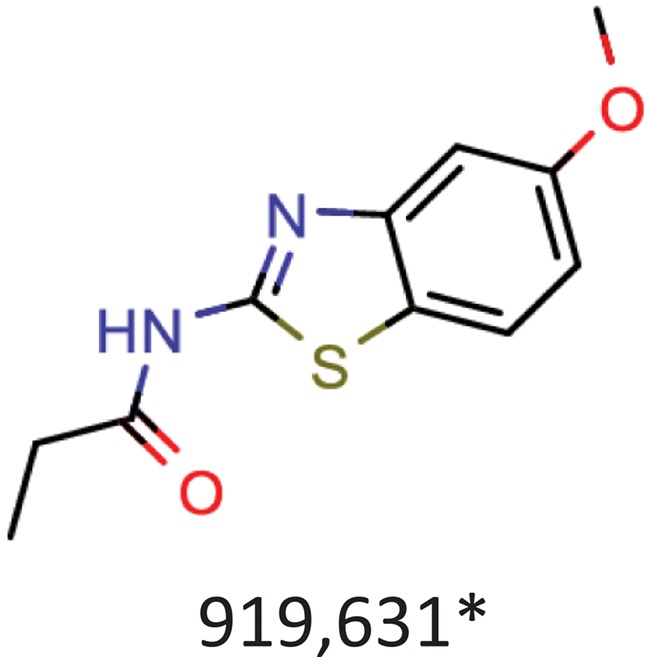	Hepatitis C virus reproduction inhibitor CHEMBL2114775^**^	0.113	0.7080	0.8071
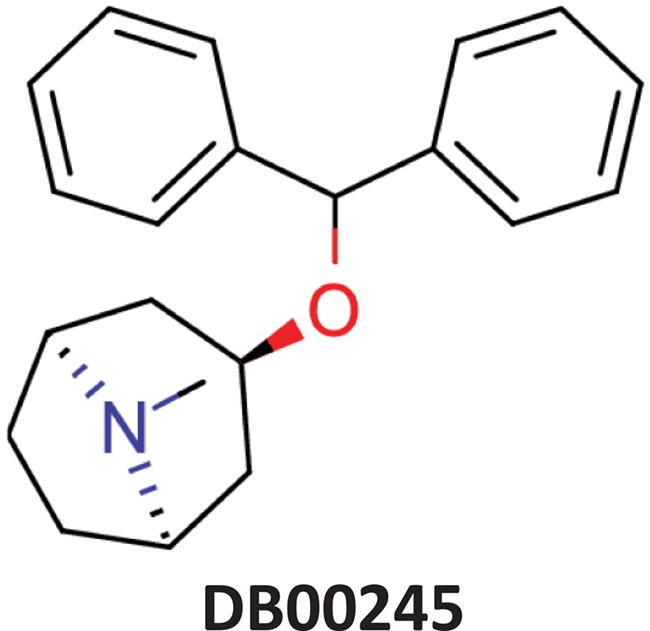	Benztropine	Parkinsonism; control of extrapyramidal disorders due to neuroleptic drugs.	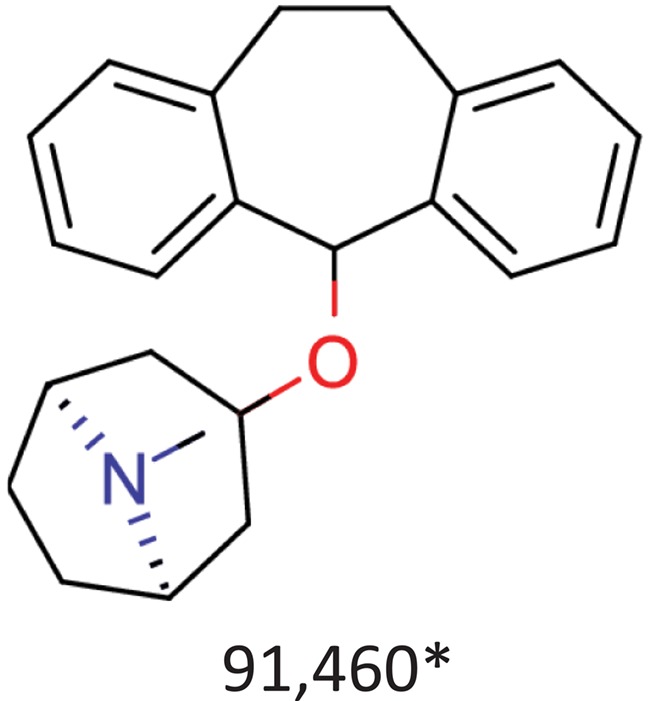	Entry inhibitor for Lassa virusCHEMBL1794308^**^	0.148	1.0226	0.8861

^*^ stdstr_id from stdstr_mrgn table

^**^CHEMBL ID of the assay

### Antiviral chemical space

SOMs were used as a simple approach to visualize the antiviral chemical space represented by ViralChEMBL (Figure 7a). ViralChEMBL compounds are diverse and occupy most regions of the map. To illustrate our data enhancement, we colored the SOM dots according to the viral family against which a compound was tested (Figure 7b). This taxonomy level was used as a compromise between the number of different colors to be used on the map and information content of the plot.

It may be easily observed that most compounds were tested against single families. As usual, most of them correspond to families *Retroviridae* (the most studied member is *HIV-1*), *Flaviviridae* (*Hepatitis C virus*) and *Orthomyxoviridae* (*Influenza virus A*). Several compound classes, usually explored against certain viruses, form tight clusters. Compounds tested against multiple virus families are small in number and scattered around the map.

For each of 158 virus species with interpretable activity data, the map can be colored by activity. However, visual analysis is easy only for species with rather a high number of data points (Figure 7c–e). Enrichment of *HIV-1 (*Figure 7c), *Hepatitis C virus* (Figure 7d) and *Influenza A virus* (Figure 7e) maps with actives clearly shows the publication bias in literature-based data; on the contrary, data for less studied *Dengue virus* (Figure 7f) are often published along with the data for other viruses, and activity is observed only against one of them.

Maps of antiviral chemical space may be useful to identify the possible antiviral activity of new or repurposed compounds. As a simple example, we projected DrugBank onto the ViralChEMBL SOM (Figure 8) and used SOM distance between pairs of compounds as an alternative to Tanimoto similarity for searching compound pairs. Two examples of such pairs are given in Table 11. Further elaborated studies for antiviral activity prediction may be developed based on ViralChEMBL data.

## Conclusions & Future Directions

Discovery of new antiviral drugs is a very important problem of medicinal chemistry, justified by the emergence of novel viruses and resistance of known ones. A large amount of antiviral activity data is available in the most widely used public repository ChEMBL, but these data require additional annotation to be used for the mapping of antiviral chemical space. To overcome this problem, we developed an algorithm of semi-automatic curation of ChEMBL data based on mapping lists for assay organism and target organism data and dictionary of virus-related terms. The work of this algorithm was demonstrated using ChEMBL 20 and ICTV taxonomy 2014 by a generation of the first version of antiviral activity data set ViralChEMBL, available as Supplementary data 17 to this paper. SQL version of the database used for the data management is provided as Supplementary data 18.

Both ChEMBL database and ICTV taxonomy are not stable entities, subject to change due to science development. As for now, ViralChEMBL presents just a snapshot of both data systems. A convenient approach for updating of the database along with ChEMBL and ICTV taxonomy is being developed now. In this paper, we describe the development of general procedures for efficient extraction of antiviral activity data from public databases. These procedures were applied to ChEMBL release 20 that was current at the time of the start of the work. The major aim of the study was to demonstrate the data handling workflow and its applicability to a real data set, as well as to provide some simple analysis of the antiviral chemical space as an illustration. Automated procedures of antiviral activity database generation are developed now, and a web server implementation will be prepared for ViralChEMBL to make the analysis of the antiviral chemical space more accessible for the community.

## Author contributions

A.A.N., A.A.O. and D.I.O. designed the study. A.A.N. and A.A.O. developed and coded the algorithms. A.A.N., A.A.O. and D.I.O. extracted and checked the data. L.I.K. supervised and checked virology-related issues. V.A.P. supervised the study. D.I.O., A.A.O. and A.A.N. wrote the manuscript. All authors have commented and approved the final manuscript.

## Availability of data and material

The data sets supporting the conclusions of this article are included within the article (and its additional files).

## Supplementary Material

Supplementary Data

## Notes

**Database URL**: http://viraldb.org
