# Supplementary material for: Enhanced taxonomy annotation of antiviral activity data from ChEMBL
Source: Database (Oxford). 2019 Feb 8;2019:bay139. doi: 10.1093/database/bay139 (PMC6367519; doi:10.1093/database/bay139)
Supplement: Supplementary Data [file bay139_supp.zip › Supplementary Information.docx]

# Supplementary Information

Supplementary File 1. Database schema used for ViralChEMBL dataset processing. Taxonomy tables are colored green, compound/species statistics - purple, tables used for data processing - blue, tables extracted from initial ChEMBL schema - grey. ‘ICTV MSL’ - ICTV Master Species List v. 2014; ‘bioactivity-16_12_04_18’ - virus-related data extracted with Taxonomy Browser interface of ChEMBL website (https://www.ebi.ac.uk/chembl/target/browser).

Supplementary File 2. List of pathogenic virus species.

Supplementary File 3. List of virus-related values of assays.assay_organism*.*

Supplementary File 4. List of virus-related values of target_dictionary.organism*.*

Supplementary File 5. Dictionary of virus names and name variants.

Supplementary File 6. Script for antiviral assay data extraction.

Supplementary File 7. Standardizer XML file.

Supplementary File 8. Summary of activity types sorted by the number of records in the database.

Supplementary File 9. Script for activity data standardization.

Supplementary File 10. List of substring—species_id pairs.

Supplementary File 11. Dictionary of pairs of virus names containing common substring.

Supplementary File 12. Script for antiviral assays data mapping.

Supplementary File 13. Script for final taxonomy identifier assignment.

Supplementary File 14. Testing heatmap. Vertical axis: virus species. Horizontal axis: molecular weight. Each pixel comprises 1,362 compounds.

Supplementary File 15. Comparative analysis of functional groups in ViralChEMBL vs ChEMBL databases.

Supplementary File 16. Color legend for Figure 7b.

Supplementary File 17. ViralChEMBL dataset.

Supplementary File 18. SQL-version of database used for data management.
