# Supplementary figures and images for: Enhanced taxonomy annotation of antiviral activity data from ChEMBL
Source: Database (Oxford). 2019 Feb 8;2019:bay139. doi: 10.1093/database/bay139 (PMC6367519; doi:10.1093/database/bay139)

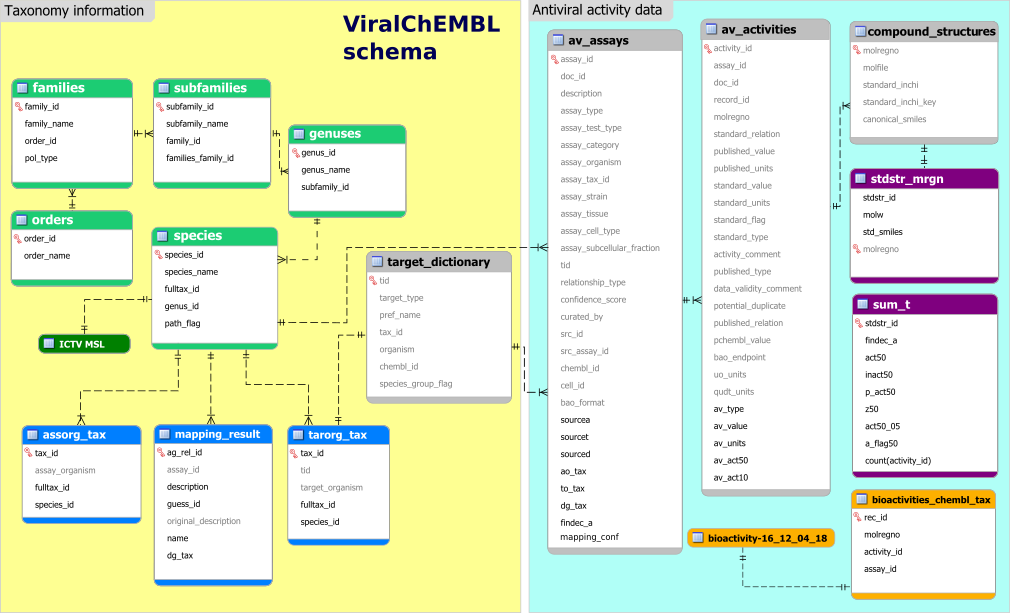

Supplement: Supplementary Data [file bay139_supp.zip › SF01_ViralChEMBL_schema.png]

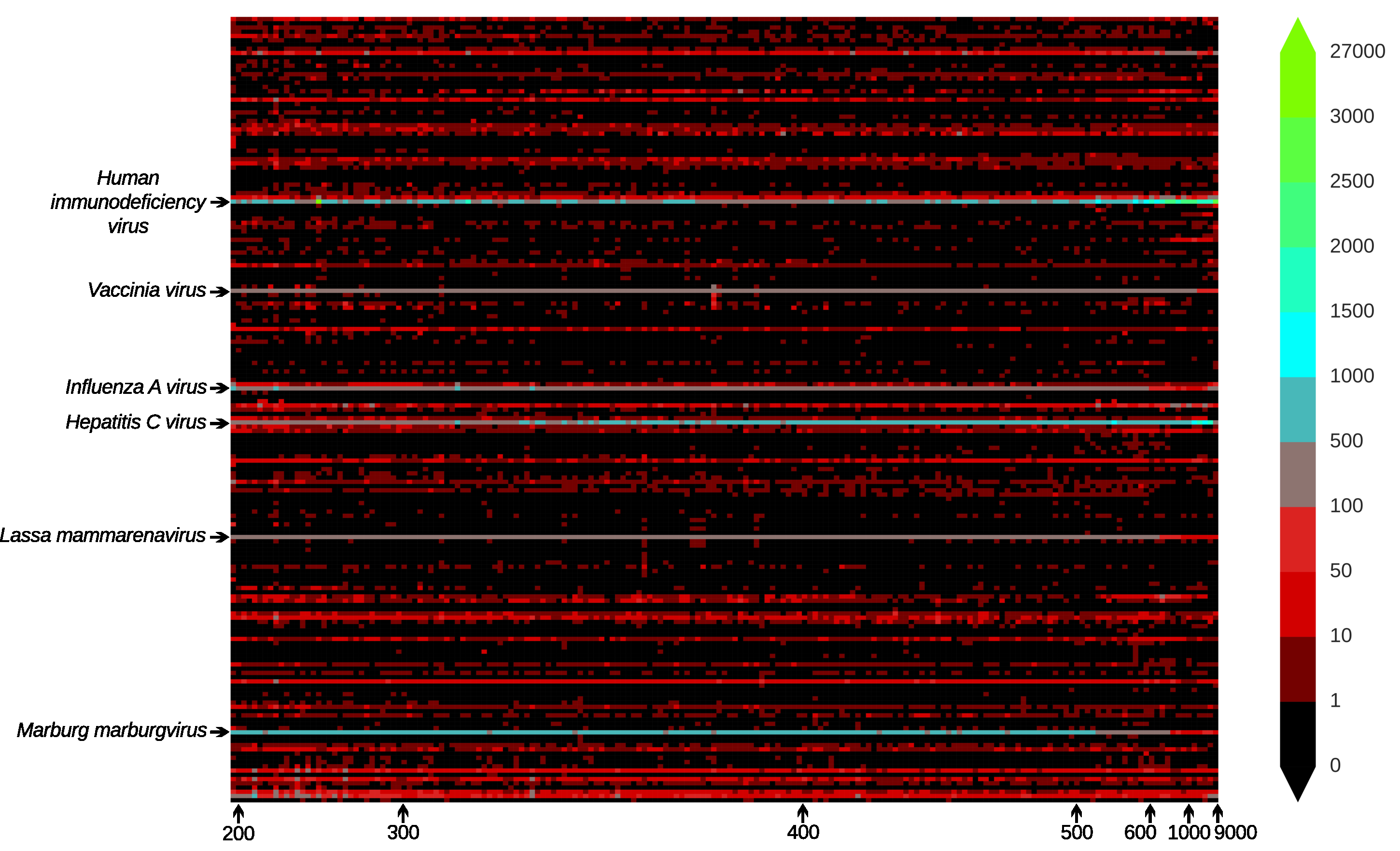

Supplement: Supplementary Data [file bay139_supp.zip › SF14_testing_heatmap.tiff]

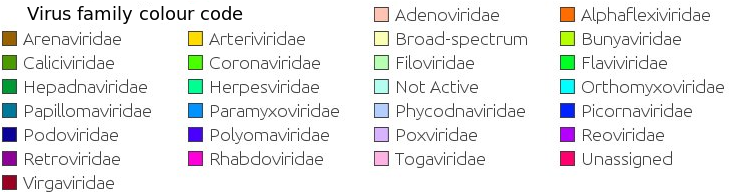

Supplement: Supplementary Data [file bay139_supp.zip › SF16_Figure7b_ColourLegend.tif]
